# Understorey bird assemblages in selected environmentally sensitive areas (ESA) of Selangor, Peninsular Malaysia

**DOI:** 10.3897/BDJ.11.e95670

**Published:** 2023-04-05

**Authors:** Kaviarasu Munian, Nur Aina Amira Mahyudin, Shahfiz Mohammad Azman

**Affiliations:** 1 Zoology Branch, Forest Biodiversity Division, Forest Research Institute Malaysia (FRIM), 52109, Kepong, Selangor Darul Ehsan, Malaysia Zoology Branch, Forest Biodiversity Division, Forest Research Institute Malaysia (FRIM) 52109, Kepong, Selangor Darul Ehsan Malaysia; 2 Environmental Management and Conservation Research Unit (eNCORe), Faculty of Applied Sciences and Technology, Universiti Tun Hussein Onn Malaysia (Pagoh Campus), 84000, Muar, Johor Darul Ta'zim, Malaysia Environmental Management and Conservation Research Unit (eNCORe), Faculty of Applied Sciences and Technology, Universiti Tun Hussein Onn Malaysia (Pagoh Campus) 84000, Muar, Johor Darul Ta'zim Malaysia

**Keywords:** avifauna, biomass, conservation, diversity, permanent forest reserves

## Abstract

Environmentally Sensitive Areas (ESA) refer to areas that are of critical importance in terms of ecosystem services such as goods, services and life-support systems, such as water purification, pest control and erosion regulation. In addition, they also refer to areas that harbour the wealth of the nation’s biodiversity. However, the classification of ESA in Malaysia is incomprehensible and lacks weightage on biological elements as the current classification is more centred on physical attributes. In order to enhance the existing classification of ESA by introducing biological elements, biological data are urgently required, especially for forest reserves and protected habitat. Hence, we conducted understorey birds surveys in three ESA rank II permanent forest reserves, located in northern Selangor as baseline information to strengthen the ESA classification. The surveys were carried out using mist-netting in three 400 m × 200 m plots. Alpha diversity indices were calculated and showed a significant difference in terms of diversity, composition and biomass of understorey birds between investigated sites. Analysis of similarity (ANOSIM) showed that bird assemblages from forest reserves designated as ESA rank II in Selangor, based on disturbances levels, have weakly diverged and SIMPER analysis has identified six species that contributed to 60% of the differences amongst the bird assemblages. The finding provides the first insight into understorey birds of the study sites and the importance of conserving and preserving ESA of permanent forest reserves, especially the small and fragmented forests.

## Introduction

Anthropogenic disturbances resulting in habitat loss, reduction and extinction of biodiversity impose immense pressures on the integrity of natural ecosystems and jeopardise the quality of basic needs, such as clean air and water (Herrera-Silveira and Morales-Ojeda 2009, Dearborn and Kark 2010). Comprehensive landscape planning is vital to create a quality environment as most of the ecosystem services are dependent on the land cover which is influenced, to a large extent, by land use (Festus 2014). Negative impacts induced by human activities should be mitigated urgently with integrated approaches and techniques to ensure the sustainable use of natural resources and functions of ecological services (Ignatieva et al. 2010). One of the land-use-based approaches that are being practised worldwide is the implementation of Environmentally Sensitive Areas (ESA). This concept was first introduced in the United Kingdom through the Agriculture Act 1986, where specific environments of national interest, such as areas of importance to environmental health and areas threatened by farming practices (MAFF 1989) were targeted. In the USA, ESA refers to a piece of land set aside to protect particular natural environments, such as recreational areas, wilderness areas, wildlife refuges and historic sites (Watson et al. 1995).

Malaysia employs a similar ESA approach and its definition depends on the type of governance framework. The Department of Environment (DoE) describes ESA as an area that requires special attention before the approval of development in a particular place and adjacent areas (Jabatan Alam Sekitar 1993). The Department of Town and Country Planning Peninsular Malaysia (PLANMalaysia) expounds ESAs from a land-use planning perspective and defines them as “…a special area that is very sensitive to any form of change to its ecosystem due to natural processes or activities in or around it, either directly or indirectly, where its level of sensitivity is determined based on the integration of elemental features like disaster risk, life support value as well as the value of the area's natural treasure and heritage” (Jabatan Perancangan Bandar dan Desa 2017). In 1998, the National Physical Plan identified ten categories of landscapes that fall under ESA and these include forest reserves, highlands and slopes, catchment areas and wildlife protection (Jabatan Perancangan Bandar dan Desa 1998), thus forming an integrated network of ESAs with its major functions being the provision of life support services and heritage values, as well as risk-associated hazards. Implementation of ESA in Malaysia became mandatory with the Second and Third National Physical Plans (NPP) requiring each State in the Peninsular to identify such areas in their respective jurisdiction in order to ensure more sustainable development. ESAs under NPP has three ranks, i.e. ESA Rank I, II and III (Table 1). Its implementation is guided by a comprehensive set of guidelines for the conservation and development planning issued for each of the ten categories (Jabatan Perancangan Bandar dan Desa 2017). Arising from this, all the States in the Peninsular have included ESAs in their State Structural Plan and local plans.

The identification of the Malaysian ESA is primarily based on physical attributes, such as degree of slope, elevation and risk of hazards with no biological or ecological component included (Shahfiz et al. 2021). Clearly, there is a need to improve this glaring omission, in order to meet its prescribed aim. When considering the inclusion of biological and ecological components for strengthening the ESA classification, data on species diversity, abundance, distribution, species composition or types of assemblages and threat status are the logical first requirements. Yet, such biological information in Malaysia is still scarce and requires continuous documentation on various aspects of biological components. Hence, this study was conducted on bird diversity in the State of Selangor, aiming to establish such data. Birds are essential to the ecosystem because they serve as pollinators and seed dispersers (Nason 1992). Furthermore, birds are good predictors of the current state of the forests' well-being (Zakaria et al. 2005) including disturbance (Barlow et al. 2006), floral composition and food availability because they are highly sensitive to changes in vegetation structure and composition (Barlow et al. 2006, Zakaria et al. 2013). Furthermore, they can signify long-term environmental disturbances, such as urbanisation, air pollution and landscape alteration (Sidra et al. 2015).

The State of Selangor, being the most populous and advanced state in Peninsular Malaysia, is home to a remarkable number of bird species, accounting for 74% of the total bird species found in the entire country. Amongst these bird species, 38 are globally-threatened, including the Mountain Peacock-pheasant (*Polyplectroninopinatum*), Short-toed Coucal (*Centropusrectunguis*), Masked Finfoot (*Heliopaispersonatus*) and Helmeted Hornbill (*Rhinoplaxvigil*), as identified by Clements et al. (2021). As of 2021, the estimated human population of Selangor is approximately 6.5 million. With a high rate of urbanisation, Selangor has witnessed significant development, including high-rise buildings, highways and industrial complexes. Hence, there is a potential risk of significant impact on the biodiversity within the State. High-paced developments can cause habitat loss, fragmentation and degradation, which can lead to a decline in the number and diversity of species. Therefore, it is important to implement effective conservation measures and sustainable development practices to mitigate the potential negative impacts of these developments on the biodiversity of Selangor, especially on the bird diversity. However, data on the bird diversity, composition and distribution are still scarce across the forest reserves in Selangor. To initiate the inclusion of biodiversity into ESA, this understorey bird study aimed to: (1) document the species richness in three ESA sites within Selangor; (2) compare the diversity, composition and biomass of bird assemblages between ESAs; and (3) investigate the differences in bird assemblages with other ESA Forest Reserves in Selangor, based on land-use changes.

## Materials and Method

**Study Sites**

For the present study, three ESA sites were chosen - Bukit Kutu Forest Reserve (BKFR), Gading Forest Reserve (GFR) and Bukit Tarek Forest Reserve Extension (BTE). These sites are lowland tropical rainforests that have an elevation range of 100 m up to 1650 m above sea level. The sites selected for the present study are in ESA Rank II under the State Structural Plan of Selangor 2020.

Amongst the selected sites, Bukit Kutu Forest Reserve (BKFR) is situated along the Titiwangsa Range and is surrounded by other forest reserves like Semangkok FR and Batang Kali FR. It covers an area of 6,452 ha of lowland and hill dipterocarp forest with an elevation ranging from 250-1053 m a.s.l. Established as a wildlife reserve in 1992, it was later gazetted as a part of the Selangor State Park (SSP). BKFR is a popular destination for hikers and visitors and there are a few Orang Asal villages located at its entry. The study plot in BKFR comprises a mixture of forest trees and fruit trees, such as durians (*Durio* spp.), jackfruit (*Artocarpusheterophyllus*), mangosteen (*Garciniamangostana*) and rambutan (*Nepheliumlappaceum*) that are cultivated for sale.

Another selected site, Gading Forest Reserve (GFR), is one of the largest forest complexes in Selangor and is situated along the Titiwangsa Range. It covers an area of about 19,034.8 ha and the highest peak is about 1650 m. The plots in GFR and BKFR are located approximately 20 km apart and connected via Semangkok Forest Reserve (west-south of GFR). GFR is double-gazetted as a part of the Selangor State Park and is an important water catchment area that is entirely protected. GFR is predominantly covered with dense and matured vegetation of lowland and hill dipterocarps. There is no development or agriculture activity within a 0.5 km radius of the study plot.

**Figure 1.**
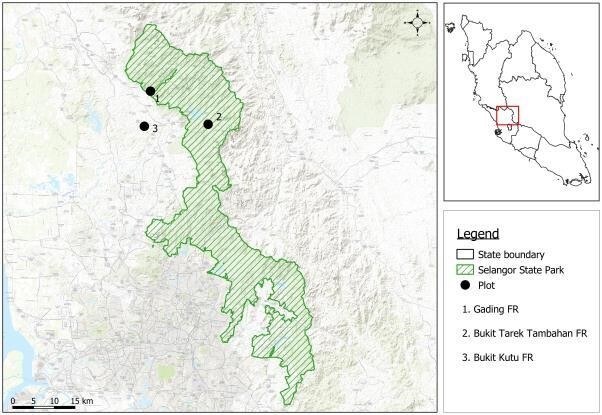
Locations of study sites of understorey birds in Gading Forest Reserve, Bukit Kutu Forest Reserve and Bukit Tarek Forest Reserve (E).

Finally, Bukit Tarek Forest Reserve Extension (BTE) abuts Bukit Tarek FR and is located 10 km south of GFR and 15 km west of BKFR. It covers an area of 3,560 ha of forest that is significantly fragmented and surrounded by rubber and palm-oil plantations. Unlike BKFR and GFR, BTE is located outside the Selangor State Park (Fig. 1).

### Understorey bird inventory

We defined understorey as the strata under the forest canopy with height from forest ground up to 5-6 m. We conducted the understorey bird inventory from early 2016 until April 2019. A total of ten mist-nets sized (12 × 2.5 m) were deployed in a 400 × 200 m plot in each study site in the respective forest reserve. Each sampling session was conducted for five consecutive days (duration) and a total of seven sampling sessions were carried out within each plot. Each mist-net was fixed to a pair of collapsible poles with heights of 3-5 m. All the nets were fixed at potential fly paths within the plot. Each net was checked every two hours starting from 06:30 to 11:00 hours and then from 19:30 to 22:30 hours daily. The total effort for the mist-net was 2,800 net hours per site. All captured birds were carefully removed from the mist-net and temporarily placed in a cloth bag prior examination.

Then, the captured birds were measured morphologically and weighed, identified up to species level, photographed and released back to the point of capture to reduce disturbance of their daily routines. The recorded measurements were tarsus length, bill length, bill width, bill depth, head bill, total length, tail length, wing length, wingspan and weight body. Bird classification and nomenclature follow Jeyarajasingam and Pearson (2012) and Robson (2020). Several specimens were curated representing each species that were recorded. The specimens were stored in 70% ethanol and deposited into the Zoological Collection of Forest Research Institute Malaysia (FRIM), Kuala Lumpur. This research was approved by the Department of Wildlife and Parks (DWNP) Peninsular Malaysia under research permit P9.2/21/2023.

### Species diversity, composition and biomass

An individual-based rarefaction curve was plotted for the three study sites to determine the completeness of the sampling efficiency (Gotelli and Colwell 2001). We chose an individual-based- instead of a sample-based approach because our primary interest was to estimate and compare species richness (the total number of species at a particular site) rather than species density (the number of species per unit area) (Colwell et al. 2012).

We calculated and compared species diversity for understorey birds in the three sites using four different indices, namely, species richness, Shannon-Wiener diversity, Evenness and Dominance. We used the Chao 1 estimator to evaluate the total species richness expected in an area which includes species that are not caught during the survey in each study site. We also conducted t-test analysis for Shannon-Wiener and Simpson indices to explain the differences in species composition between sites.

To compare the biomass of understorey birds amongst study sites, the birds were first categorised into three trophic guilds, namely omnivorous, insectivorous and frugivorous. Then, we multiplied the mean live weight of each species with the number of individuals found in the respective sites (Johnson et al. 2011).

### Comparison of understorey bird assemblage

We compared the current findings with other bird assemblages from four other Forest Reserves in Selangor. These Reserves are ESA Rank II in the Selangor State Structural Plan (SSP). We categorised each Reserve based on the types of human activities that occur in and adjacent to the Reserves. The highest level of disturbance was given to BTE and Bukit Broga FR, followed by BKFR and Sg Lalang FR in decreasing order (Table 2). The presence/absence data for birds in these four sites were acquired from secondary sources. An analysis of similarity (ANOSIM), based on presence/absence, was performed to test the patterns of species composition amongst the seven Forest Reserves, based on three categories. The ANOSIM procedure is a non-parametric permutation test that is analogous to an ANOVA for similarity matrices (Clarke and Warwick 2001) to test whether predefined classes differ in mean similarities/dissimilarities. A similarity percentage (SIMPER) analysis was also used to examine the contribution of each species towards the differences detected in the comparison between the Forest Reserves. All the analysis were conducted using R package Vegan (Oksanen et al. 2019) and iNext Package (Chao et al. 2014, Hsieh et al. 2020) in Rstudio platform (RStudio Team 2021).

## Results

### Species diversity, composition and biomass

A total of 225 individuals, from 67 species and 23 families, were captured with the highest number recorded in GFR (131 individuals from 46 species), followed by BTE (54 individuals from 33 species) and BKFR (40 individuals from 22 species) (Table 3). Of the 67 species recorded, two species were categorised as Vulnerable (VU), 13 were Near Threatened (NT) and the rest were Least Concern (LC) under the IUCN Red List of Threatened Species (IUCN 2022).

Based on the Shannon-Wiener index, GFR recorded the highest value (H = 3.43), followed by BTE with a value of H = 3.24 and BKFR gave the lowest value of H = 2.84). The index indicates that the community of understorey birds in GFR is abundant and evenly distributed amongst the species recorded compared to BKFR and BTE. Meanwhile, the understorey birds in BKFR were valued highest for Dominance (D = 0.0071) and Evenness indices (E = 0.815). The Evenness index varies from 0 (highest dominance by a single species) to 1 (all species have the same abundance) (Buzas and Hayek 2005). Interestingly, BTE recorded moderate values for the diversity indices investigated (H = 3.24, D = 0.054 and E = 0.774), even though the Forest Reserve is the most disturbed compared to other study sites (Table 4).

**Figure 2.**
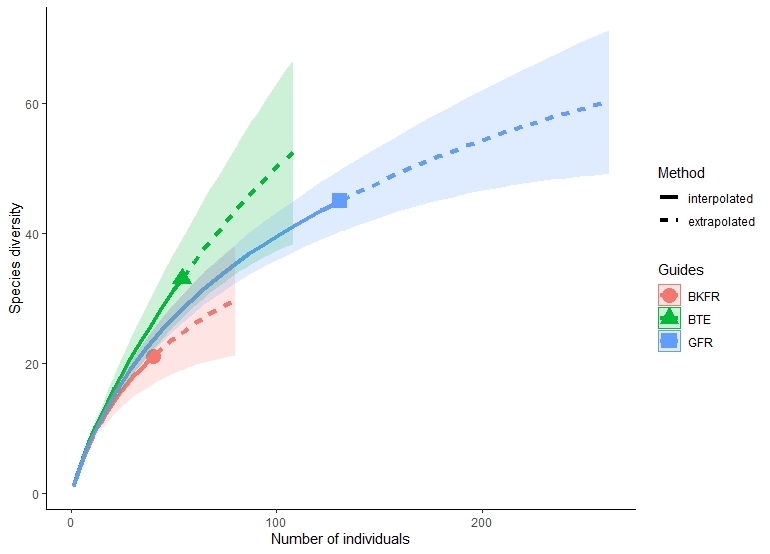
Individual-based rarefaction curves were constructed to evaluate the completeness of the survey carried out in three sites of ESA in Selangor, Malaysia and the curve revealed insufficient effort in documenting understorey birds as it had yet to reach its asymptote.

By comparison between the observed and estimated species richness (based on the Chao 1 estimator), the efforts invested in the survey only managed to recover approximately 44% to 66% of species in all three study sites. The individual-based rarefaction curve also showed that it had yet to reach its asymptote indicating the effort in documenting the understorey birds in three sites was insufficient (Fig. 2).

Species under the family Pycnonotidae were the most abundant (14 species), followed by the family Muscicapidae with nine species and Timaliidae with six species. The most abundant species was Little Spiderhunter *Arachnotheralongirostra* (10.2%), followed by Oriental Dwarf Kingfisher *Ceyxerithaca* (6.7%) and Yellow-Bellied Bulbul *Alophoixusphaeocephalus* (5.7%). There are 13 single species (consisting of 5.8%) recorded out of a total 225 individuals. One-way ANOVA indicated that the abundances of understorey birds found in three sites were significantly different (F = 6.356, df = 126.4, p = 0.00234). In GFR, Rufous-backed Dwarf Kingfisher *Ceyxerithaca* made up the largest proportion (10.6%) of total individuals captured, followed by Little Spiderhunter *Arachnotheralongirostra* (9.92%) and Yellow-Bellied Bulbul *Alophoixusphaeocephalus* (9.12%). In BKFR, Little Spiderhunter *Arachnotheralongirostra* made up 15.0% of the total individuals recorded, followed by the Green Broadbill *Calyptomenaviridis* and Grey-headed Babbler *Stachyrispoliocephala*, each with 10%. Composition of understorey birds in BTE was largely contributed by the Hairy-backed bulbul *Tricholestescriniger* (14.8%), Cream-vented Bulbul *Pycnonotussimplex* (9.2%) and Little Spiderhunter *Arachnotheralongirostra* (7.2%).

**Figure 3.**
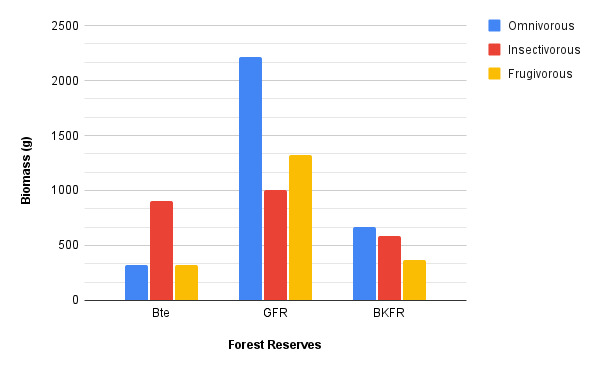
The distribution of biomass according to the omnivorous, insectivorous and frugivorous guilds in the three study sites. In general, the biomass of the omnivorous birds was the highest compared to other guilds.

The percent biomass in three trophic guilds of understorey birds varied amongst the studied sites (Fig. 3). Almost half of the understorey bird biomass in GFR was contributed by omnivorous species, 30% by frugivorous birds and only 20% constituted by insectivorous birds. Unlike in BTE, 58% of biomasses of understorey birds recorded were insectivorous birds, followed by omnivorous and frugivorous birds with approximately 29% and 22%, respectively. The biomass of understorey birds in BKFR was almost evenly distributed amongst omnivorous and insectivorous with 41% and 36%, while frugivorous birds only contributed about 22% of overall biomass in BKFR.

### Comparison between Understorey Bird Assemblages

ANOSIM analysis, based on the Bray-Curtis model, revealed a weak difference in bird assemblage composition, based on disturbances (Global R = - 0.0068, p-value = 0.483). Results of SIMPER showed that approximately 60% of the differences in assemblage composition were driven by six species, based on three levels of disturbances. They are Fluffy-backed tit babbler (*Macronusptilosus)* which contributes the highest differences (25.4%), Fiery Minivet (*Pericrocotusigneus)* and Yellow-bellied Warbler (*Abroscopussuperciliaris*)(16.7%) (Table 5).

## Discussion

**Diversity, composition and biomass of understorey birds**

Based on the method of mist-netting, we managed to document 67 (Table 3) species of understorey birds in three ESA level II permanent Forest Reserves in Selangor. To the best of our knowledge, the species compilation presented here is the first insight for bird diversity in GFR and BTE. Some studies on vertebrates were done in BKFR in 1999 (e.g. Lim et al. (1999)) and as it is a wildlife reserve, we believe that documentation on vertebrates in BKFR might be collected by the Department of Wildlife and National Park (DWNP). The compilation of birds from these three Forest Reserves would serve as baseline information for relevant authorities in making tangible measures in conserving biodiversity.

GFR recorded the highest Shannon diversity index compared to BKFR and BTE which is not entirely surprising noting that it is the largest forest complex in Selangor. The species-area relationship may explain more species richness found in GFR compared to BKFR and BTE as area increases diversity. Although the GFR was logged over 30 years ago, it appears to have the characteristics of an old growth stand due to the presence of large trees and a dense herbaceous vegetation ground layer. This could potentially provide more suitable sites for nesting and breeding, as well as a sufficient supply of food and protection from predators and harsh weather, according to studies by Reid et al. (2004), Díaz et al. (2005) and Husin and Rajpar (2015). Birds are known to be sensitive to alterations in their habitat and modifications to the landscape, as evidenced by studies by Şekercioḡlu et al. (2002), Raman (2006), Gomes et al. (2008) and Tscharntke et al. (2008).

The diversity indices indicate lowest values for BKFR compared to BTE, which is much more significantly impaired. BKFR forest is still largely covered by intact vegetation and well protected mostly due to the presence of mixed vegetation and low impact of human activities. Such results may be attributed to the survey method. This study relied on mist-netting and the height of the pole was 3-5 m above the ground. The limited height and coverage of the net were inadequate to catch understorey birds that occupy different forest strata and the taller vegetation around the plots made use of only one trapping method less effective. Another variable that could affect the results is the size and behaviour of certain understorey birds (Blake and Loiselle 2001, Wang and Finch 2002). Simultaneous use of multiple approaches such as mist-netting, spot-mapping, point counts and observation of mixed species flocks, coupled with enough sample replication and extended sampling period, could improve the results (Gram and Faaborg 1997, Herzog et al. 2002, Derlindati and Caziani 2005). Based on estimated species richness by the Chao I estimator, the effort of sampling in the study only managed to document about 60% of the diversity in the study sites. With a much longer period of sampling, the chances to document almost the actual diversity of the understorey birds would be increased. This was proven by local studies conducted in Peninsular Malaysia with prolonged periods of sampling (Johns 1996, Lambert 1997, Peh et al. 2005). Amongst the three sites, the BKFR had the most uniform spread of understorey birds. High species evenness residing in an ecosystem are represented by almost an equivalent proportion of all the species presented. The value of the Evenness index of almost one (E = 0.815) shows that the BKFR is ecologically stable in providing a vast range families of understorey birds inhabiting and surviving successfully.

The family Pycnonotidae (bulbuls) had the highest number of species recorded in all study sites. A similar trend was also reported by Mansor and Sah (2012), Nor Hashim and Ramli (2013), Rajpar and Zakaria (2014), Barlow and Peres (2016) and Shafie et al. (2018). Members of the Pycnonotidae family dominated the understorey habitat of Malaysia's tropical forest. They are generalist frugivorous species that are ecologically diverse and occupy a wide array of habitats (Ponpituk et al. 2020). The Bulbuls are particularly important in the Asian region for their role in seed dispersal, especially in habitats that are degraded (Sankamethawee et al. 2011, Corlett 2017, Shakya and Sheldon 2017). They have a high tolerance to temperature and light intensity, are well-known colonisers and prefer to inhabit logged-over forests (Mohd-Taib et al. 2018). The comparative abundance of this family in all three Reserves, in particular BTE, is an advantage as it is likely to play a key role in the regeneration and recovery of vegetation through seed dispersal.

The assemblages in the three study sites were dominated by Little Spiderhunter (*Arachnotheralongirostra*), Rufous-backed Kingfisher (*Ceyxerithaca*) and Yellow-Bellied Bulbul (*Alophoixusphaeocephalus*). The presence of Little Spiderhunter is common in tropical secondary forests where wild bananas (Musaceae) and gingers (Zingiberaceae) flourish and are eaten by birds (Nor Hashim and Ramli 2013). Little Spiderhunter is recorded from a variety of different habitats that provide a wide range of food sources, microhabitats and refuge from predation (Khan et al. 2008). The presence of Rufous-backed Kingfisher is largely linked to the presence of waterbodies, such as lakes and rivers. All the study plots were located at average distances from either small streams or fast-flowing rivers. The diet of Kingfisher mainly comprised small fishes, insects and other higher taxa bird species including frogs. The presence of a large network of rivers in the GFR directly contributes to the relatively high abundance of Rufous-backed Kingfisher compared to BTE, where only a moderately-sized stream flows through the Reserve. Yellow-bellied Bulbul is a generalist frugivorous occupying a vast range of habitats, such as pristine forests (Nor Hashim and Ramli 2013), secondary forests (Husin and Rajpar 2015), wetlands (Biun and Buang 2014) and palm-oil plantations (Amit et al. 2014).

The species compositions between study sites are significantly different as shown by ANOVA analysis. Despite the majority composition was contributed by Little Spiderhunter *Arachnotheralongirostra*, Rufous-backed Kingfisher *Ceyxerithaca* and Yellow-Bellied Bulbul *Alophoixusphaeocephalus*, BKFR was also presented by the Green Broadbill *Calyptomenaviridis* and Grey-headed Babbler *Stachyrispoliocephala*, both of which species were absent in BTE. Presence of these species indicates the evenness of composition of the understorey birds in BKFR. Additionally, these species are sensitive and mainly recorded in primary forests (Ramly and Ramli 2009, Bing et al. 2013, Mohd-Taib et al. 2018) in Peninsular Malaysia suggesting that BKFR is capable of sheltering a wide range of understorey birds. While, in BTE, Hairy-backed bulbul *Tricholestescriniger* and Cream-vented Bulbul *Pycnonotussimplex* dominated the habitat.

The biomass of understorey birds differed considerably amongst the three study sites. Overall, GFR has the largest abundance of understorey birds, followed by BKFR and BTE. Nevertheless, in the aspect of trophic guilds, the study shows that frugivorous birds were abundantly distributed compared to insectivorous and omnivorous birds. Similarly, the biomass of frugivorous birds in GFR was the highest compared to insectivorous and frugivorous. Frugivorous birds primarily feed on fruits such as figs, berries and fleshy fruits, although it was observed that many of these birds supplemented their diet with other animals, mainly insects. By comparison, GFR is denser in terms of vegetation and least impaired from disturbances. Morante-Filho et al. (2018) hypothesised that assemblages of frugivorous birds were affected by two factors: vegetation complexity and fruit availability. Habitats that are covered with more heterogeneous vegetation potentially provide more niches and offer more diverse ways to exploit different resources, such as nesting sites and shelter, while greater availability of resources engender higher species richness (Davies et al. 2007, Ferger et al. 2014). As the sampling period did not coincide with the fruiting season, the vegetation complexity might contribute to higher frugivorous birds in GFR.

The biomass in BTE was dominated by insectivorous birds. BTE is bordered by plantations of palm oil and rubber. In fact, insectivorous birds are sensitive to habitat changes and disturbances were severally reported by studies in Malaysia (e.g. Moradi et al. (2008), Mansor and Sah (2012)). A high biomass of insectivorous birds was obtained in BTE as most of the mist-nets were placed inside the forest and not near to the forest edge or next to the areas occupied by the plantations. The study by Mansor and Sah (2012) identified that the higher density of insectivore understorey birds was confined to the forest interior compared to the forest edge. Apart from that, the availability of food resources in BTE might contribute to the high occurrence of insectivorous birds with the addition of more populations of invertebrates (e.g. insects) found in areas of palm oil and rubber plantations. Other trophic guilds, such as carnivores and piscivores were primarily represented by predator species such as eagles, owls and kingfishers. Broadly, the biomass of other guild birds was the lowest as these predators were present in low numbers. They are the top predators in the food chain (i.e. tertiary consumers); thus, their populations always remain low compared to those of the primary and secondary consumers in the ecosystem (Rajpar and Zakaria 2014).

**Comparison assemblage of understorey birds**

All the Forest Reserves included in the comparison were gazetted as ESA level II of Permanent Forest Reserve under the Selangor Strategic Plan. Surprisingly, many of these Forest Reserves were logged (more than 30 years ago) and some were very recently cleared or open for development (mostly for palm oil plantations). Based on the ANOSIM analysis, there were no significant differences between the levels of disturbances investigated. The lack of differences shown by ANOSIM indicates that the level of disturbances in the seven Forest Reserves did not influence the distribution of bird composition, bearing in mind that some Reserves, such as BTE and Bukit Broga FR, are fragmented forest patches.

The negative value of ANOSIM was largely contributed by the differences in composition within an assigned group and less so by the differences between groups. This is because the information derived from published studies was subjected to distinct methods (such as point count and direct observation) apart from using mist-netting which directly reflected the richness of birds found at each study site. Nevertheless, a distinct composition of birds at group level showed that the bird assemblages were much influenced by factors, such as microhabitat, vegetation heterogeneity and food resources. Hence, it might be appropriate to conclude that each forest reserve or habitat preserves its own diversity, regardless of landscape changes occurring within the habitat.

## Conclusion

The study presented the first information on understorey birds in Bukit Tarek Forest Reserve Extension and Gading Forest Reserve together with previous research in Bukit Kutu Forest Reserve, all three of which were designated as Environmentally Sensitive Areas (ESA) Rank II in Selangor. A total of 67 species of avifauna were recorded with the highest diversity found in Gading Forest Reserve. The information collected through this study should partly serve as baseline information for developing biological attributes to be included in the ESA classification. Apart from that, the results could be used by the relevant authorities and stakeholders in managing these forest reserves soundly, based on scientific decisions and to ensure the preservation of biodiversity of avifauna.

## Figures and Tables

**Table 1.** ESA ranks based on the 2^nd^ National Physical Plan of Malaysia.

**Rank**	**Descriptions**
ESA Rank I	No development, agriculture or logging shall be permitted, except for low-impact nature tourism, research and education.
ESA Rank II	No development or agriculture. Sustainable logging and low impact nature tourism may be permitted subject to local constraints.
ESA Rank III	Controlled development whereby the type and intensity of the development shall be strictly controlled depending on the nature of the constraints.

**Table 2.** The locations and details of three selected environmentally sensitive areas and additional forest reserves located in Selangor. The level of disturbance in study sites and additional forest reserves were categorised, based on following four major activities: 1 = villages, 2 = hiking/tourist spot, 3 = mixture vegetation and 4 = oil palm/rubber plantation.

**Sites**	**Coordinates**	**Study**	**Activities**				**Level of Disturbance**
			**1**	**2**	**3**	**4**	
GFR	3°37'43.35"N, 101°37'16.64"E	Present Study					Fair
BTE	3°31'22.86"N, 101°36'27.00"E		√		√	√	Poor
BKFR	3°33'20.8"N, 101°44'19.5"E			√	√		Mild
Sg Lalang FR	2°57'N, 101°54'09"E	Lim et al. (2009)	√	√			Mild
Sg Congkak Recreational Forest	3°12'42.32"N, 101°50'36.46"E	Bakri et al. (2016)		√			Fair
Bukit Broga FR	2°57'N, 101°54'09"E	Lim et al. (2009)		√	√	√	Poor
Hulu Langat FR (Pangsun and Gunung Nuang)	3°13'N, 101°52'E	Shafawati and Md-Nor (2009)	√	√			Mild

**Table 3.** Understorey bird diversity and numbers recorded in GFR, BTE and BKFR and their IUCN Red List status. Birds from additional four Forest Reserves were indicated based on presence and absence data (X/-=presence/absence). SCFR= Sg Congkak Recreational Forest, BBFR= Bukit Broga Forest Reserve, SLFR= Sg Lalang Forest Reserve and HLFR= Hulu Langat Forest Reserve (Pangsun and Gunung Nuang).

**Species**	**Common Name**	**BTE**	**GFR**	**BKFR**	**SCRF**	**BBFR**	**SLFR**	**HLFR**	**IUCN Status**
Accipitriformes									
*Spilornischeela*	Crested Serpent-eagle	0	0	0	-	X	X	-	LC
*Nisaetuscirrhatus*	Changeable Hawk-eagle	0	0	0	-	-	X	-	LC
*Nisaetusalboniger*	Blyth's Hawk-eagle	0	0	0	-	-	X	-	LC
*Accipitergularis*	Japanese Sparrowhawk	0	0	0	-	-	-	-	LC
Bucerotiformes									
Bucerotidae									
*Bucerosrhinoceros*	Rhinoceros Hornbill	0	0	0	-	X	X	-	VU
*Rhinoplaxvigil*	Helmeted Hornbill	0	0	0	-	-	X	-	CR
*Anorrhinusgaleritus*	Bushy-crested Hornbill	0	0	0	-	-	X	-	NT
*Berenicorniscomatus*	White-crowmed Hornbill	0	0	0	-	-	X	-	EN
*Rhabdotorrhinuscorrugatus*	Wrinkled Hornbill	0	0	0	-	-	X	-	VU
Caprimulgiformes									
Apodidae									
*Apusaffinis*	Little Swift	0	0	0	-	X	X	-	LC
*Rhaphiduraleucopygialis*	Silver-rumped Spinetail	0	0	0	-	X	X	-	LC
*Cypsiurusbalasiensis*	Asian Palm-swift	0	0	0	-	X	X	-	LC
Caprimulgidae									
*Lyncornistemminckii*	Malay Eared-nightjar	1	0	0	-	-	-	-	LC
*Caprimulgusmacrurus*	Large-tailed Nightjar	0	0	0	-	X	-	-	LC
*Caprimulgusaffinis*	Savanna Nightjar	1	0	0	-	-	-	-	LC
Hemiprocnidae									
*Hemiprocnelongipennis*	Grey-rumped Treeswift	0	0	0	-	X	X	-	LC
*Hemiprocnecomata*	Whiskered Treeswift	0	0	0	-	X	X	-	LC
Podargidae									
*Batrachostomusjavensis*	Horsfield's Frogmouth	0	0	0	-	-	X	-	LC
Columbiformes									
Columbidae									
*Chalcophapsindica*	Common Emerald Dove	1	6	1	X	X	X	X	LC
*Treroncurvirostra*	Thick-billed Green-pigeon	0	0	0	-	X	X	-	LC
*Treronvernans*	Pink-necked Green-pigeon	0	0	0	-	X	-	-	LC
*Macropygiaunchall*	Barred Cuckoo-dove	0	0	0	-	-	-	-	LC
Coraciiformes									
Alcedinidae									
*Alcedopeninsulae*	Malay Blue-banded Kingfisher	0	0	3	-	-	X	-	NT
*Alcedomeninting*	Blue-eared Kingfisher	1	0	0					
*Actenoidesconcretus*	Rufous-collared Kingfisher	1	1	1	X	-	X	X	NT
*Ceyxerithaca*	Rufous-backed Kingfisher	0	1	2	X	-	X	X	LC
*Lacedopulchella*	Banded Kingfisher	0	0	0	-	X	X	X	LC
*Halcyoncoromanda*	Ruddy Kingfisher	0	0	0	-	-	-	X	LC
*Todiramphuschloris*	Collared Kingfisher	0	0	0	-	-	-	X	LC
Coraciiformes									
Meropidae									
*Meropsphilippinus*	Blue-tailed Bee-eater	1	0	0	-	-	-	-	LC
*Meropsviridis*	Blue-throated Bee-eater	0	0	0	-	X	-	-	LC
*Nyctyornisamictus*	Red-bearded Bee-eater	0	0	1	-	-	X	-	LC
Cuculiformes									
Cuculidae									
*Centropussinensis*	Greater Coucal	0	0	0	-	X	X	-	LC
*Phaenicophaeuscurvirostris*	Chestnut-breasted Malkoha	1	1	0	X	-	X	-	LC
*Cacomantissepulcralis*	Rusty-breasted Cuckoo	0	0	0	-	X	X	X	LC
*Cuculusmicropterus*	Indian Cuckoo	0	0	0	-	X	X	-	LC
*Cacomantissonneratii*	Banded Bay Cuckoo	0	0	0	-	X	X	-	LC
*Cacomantismerulinus*	Plaintive Cuckoo	0	0	0	-	X	X	-	LC
*Chrysococcyxxanthorhynchus*	Violet Cuckoo	0	0	0	-	X	X	-	LC
*Surniculuslugubris*	Square-tailed Drongo-cuckoo	0	0	0	-	X	X	-	LC
*Phaenicophaeusdiardi*	Black-bellied Malkoha	0	0	0	-	-	X	-	NT
*Rhinorthachlorophaea*	Raffles's Malkoha	0	0	0	-	-	X	-	LC
*Zanclostomusjavanicus*	Red-billed Malkoha	0	0	0	-	-	X	-	LC
Falconiformes									
Falconidae									
*Microhieraxfringillarius*	Black-thighed Falconet	0	0	0	-	X	-	-	LC
*Falcoperegrinus*	Peregrine Falcon	0	0	0	-	-	-	X	LC
Galliformes									
Phasianidae									
*Gallusgallus*	Red Junglefowl	0	0	0	-	X	-	-	LC
*Argusianusargus*	Great Argus	0	0	0	-	-	X	-	NT
Gruiformes									
Rallidae									
*Amaurornisphoenicurus*	White-breasted Waterhen	0	0	0	X	-	-	X	LC
Passeriformes									
Aegithinidae									
*Aegithinaviridissima*	Green Iora	1	0	0	-	X	X	-	NT
*Aegithinalafresnayei*	Great Iora	0	0	0	-	X	X	-	LC
Alcippeidae									
*Alcippeperacensis*	Mountain Fulvetta	0	0	0	-	-	-	-	LC
*Calyptomenidae*									
*Calyptomenaviridis*	Green Broadbill	0	1	4	-	-	X	X	NT
Campephagidae									
*Pericrocotusigneus*	Fiery Minivet	0	0	0	X	X	X	-	LC
*Lalagefimbriata*	Large Cuckooshrike	0	0	0	-	-	X	-	LC
*Pericrocotusflammeus*	Scarlet Minivet	0	0	0	-	X	X	-	LC
Cisticolidae									
*Orthotomusatrogularis*	Dark-necked Tailorbird	0	1	0	X	X	X	-	LC
*Orthotomussutorius*	Common Tailorbird	0	0	0	X	X	X	-	LC
*Priniarufescens*	Rufescent Prinia	0	0	0	-	X	-	-	LC
*Orthotomussericeus*	Rufous-tailed Tailorbird	0	0	0	X	-	X	X	LC
*Orthotomusruficeps*	Ashy Tailorbird	0	0	0	X	-	-	-	LC
*Priniaflaviventris*	Yellow-bellied Prinia	1	0	0	-	X	-	-	LC
Chloropseidae									
*Chloropsiscochinchinensis*	Blue-winged Leafbird	0	0	0	X	X	X	-	NT
*Chloropsiscyanopogon*	Lesser Green Leafbird	0	0	0	X	-	X	-	NT
*Chloropsissonnerati*	Greater Green Leafbird	0	0	0	-	X	X	-	EN
Dicaeidae									
*Prionochiluspercussus*	Crimson-breasted Flowerpecker	1	3	2	-	-	X	-	LC
*Prionochilusmaculatus*	Yellow-breasted Flowerpecker	0	1	0	X	-	X	X	LC
*Dicaeumtrigonostigma*	Orange-bellied Flowerpecker	0	0	0	X	X	X	X	LC
*Dicaeumchrysorrheum*	Yellow-vented Flowerpecker	0	0	0	-	-	X	-	LC
*Dicaeumminullum*	Plain Flowerpecker	0	0	0	-	-	X	-	LC
*Dicaeumeveretti*	Brown-backed Flowerpecker	0	0	0	-	-	-	X	NT
Dicruridae									
*Dicrurusaeneus*	Bronzed Drongo	0	1	0	-	-	-	X	LC
*Dicrurusannectens*	Crow-billed Drongo	0	0	1	-	-	-	-	LC
*Dicrurusparadiseus*	Greater Racquet-tailed Drongo	2	1	1	X	X	X	-	LC
*Dicrurusmacrocercus*	Black Drongo	0	0	0	-	-	-	-	LC
*Dicrurusremifer*	Lesser Racquet-tailed Drongo	0	0	0	-	-	-	-	LC
Estrildidae									
*Lonchurastriata*	White-rumped Munia	0	0	0	X	X	-	-	LC
Eurylaimidae									
*Eurylaimusjavanicus*	Banded Broadbill	0	0	0	-	-	X	-	NT
*Eurylaimusochromalus*	Black-and-yellow Broadbill	0	0	0	-	X	X	-	NT
*Corydonsumatranus*	Dusky Broadbill	0	0	0	-	-	X	-	LC
*Psarisomusdalhousiae*	Long-tailed Broadbill	0	0	0	-	-	-	-	LC
Hirundinidae									
*Hirundorustica*	Barn Swallow	0	0	0	-	X	X	-	LC
*Hirundotahitica*	Tahiti Swallow	0	0	0	-	X	X	-	LC
Laniidae									
*Laniustigrinus*	Tiger Shrike	1	0	0	X	X	-	X	LC
Irenidae									
*Irenapuella*	Asian Fairy-bluebird	0	0	0	-	X	X	X	LC
Monarchidae									
*Terpsiphoneparadisi*	Asian Paradise Flycatcher	2	0	0	X	-	X	X	LCLC
*Hypothymisazurea*	Black-naped Monarch	0	1	0	-	-	-	X	LC
Motacillidae									
*Motacillacinerea*	Grey Wagtail	0	0	0	X	-	-	-	LC
Muscicapidae									
*Anthipessolitaris*	Rufous-browed Flycatcher	0	0	0	-	-	-	-	LC
*Copsychussaularis*	Oriental Magpie Robin	0	0	0	X	X	-	-	LC
*Cyornisbanyumas*	Hill Blue Flycatcher	0	0	0	-	-	-	X	CR
*Cyornisbrunneatus*	Brown-chested Jungle-flycatcher	0	1	0	-	-	-	-	VU
*Cyornisglaucicomans*	Chinese Blue-flycatcher	0	1	0	-	-	-	-	LC
*Cyornisrufigastra*	Mangrove Blue-flycatcher	1	0	0	-	-	-	-	LC
*Cyornismagnirostris*	Large Blue-flycatcher	1	0	0	-	-	-	-	LC
*Cyornisconcretus*	White-tailed Flycatcher	0	0	0	-	-	-	X	LC
*Cyornisrubeculoides*	Blue-throated Flycatcher	0	0	0	-	-	-	-	LC
*Cyornistickelliae*	Tickell's Blue Flycatcher	0	0	0	-	-	-	X	LC
*Ficeduladumetoria*	Rufous-chested Flycatcher	0	0	1	-	-	-	X	LC
*Ficedulamugimaki*	Mugimaki Flycatcher	0	0	0	-	-	-	X	LC
*Ficedulasuperciliaris*	Ultramarine Flycatcher	0	0	0	-	-	-	X	LC
*Larvivoracyane*	Siberian Blue Robin	3	6	1	X	-	-	X	LC
*Kittacinclamalabarica*	White-rumped Shama	1	2	0					LC
*Enicurusleschenaulti*	White-crowned Forktail	0	3	0	X	-	-	-	LC
*Enicurusruficapillus*	Chestnut-naped Forktail	0	0	0	X	-	X	X	NT
*Eumyiasthalassinus*	Verditer Flycatcher	0	0	0	-	X	X	-	LC
*Myiomelaleucura*	White-tailed Blue Robin	0	0	0	-	-	-	X	LC
*Monticolasolitarius*	Blue Rock-thrush	0	1	0	-	-	-	-	LC
*Muscicapadauurica*	Asian Brown Flycatcher	0	0	0	X	-	-	-	LC
*Muscicapasibirica*	Dark-sided Flycatcher	0	0	0	-	-	-	-	LC
*Muscicapawilliamsoni*	Brown-streaked Flycatcher	0	0	0	-	-	-	X	NE
Nectariniidae									
*Aethopygatemminckii*	Temminck's Sunbird	0	0	0	-	-	X	-	LC
*Aethopygasaturata*	Black-throated Sunbird	0	0	0	-	-	-	-	LC
*Anthreptessimplex*	Plain Sunbird	0	0	0	-	X	X	-	LC
*Anthreptesrhodolaemus*	Red-troated Sunbird	0	0	0	-	-	X	-	NT
*Arachnotheraaffinis*	Streaky-breasted Spiderhunter	0	0	0	-	X	X	X	LC
*Arachnotheraflavigaster*	Spectacled Spiderhunter	0	0	0	-	-	X	-	LC
*Arachnotherachrysogenys*	Yellow-eared Spiderhunter	0	0	0	-	-	X	-	LC
*Arachnotheramodesta*	Grey-breasted Spiderhunter	0	1	2	X	-	-	X	LC
*Arachnotheralongirostra*	Little Spiderhunter	4	13	6	X	X	X	X	LC
*Arachnotherarobusta*	Long-billed Spiderhunter	1	0	0	-	X	X	X	LC
*Chalcopariasingalensis*	Ruby-cheeked Sunbird	0	0	0	-	-	X	-	LC
*Cinnyrisjugularis*	Olive-backed Sunbird	0	0	0	-	-	-	X	LC
*Kurochkinegrammahypogrammica*	Purple-naped Sunbird	2	2	0	X	-	X	X	LC
*Leptocomabrasiliana*	Van Hasselt's Sunbird	0	1	0					LC
*Leptocomasperata*	Purple-throated Sunbird	0	1	0	-	-	-	-	LC
Oriolidae									
*Oriolusxanthonotus*	Dark-throated Oriole	0	0	0	X	-	-	-	NT
Paridae									
*Melanochlorasultanea*	Sultan Tit	0	0	0	-	-	X	-	LC
Pellorneidae									
*Malacocinclaabbotti*	Abbott's Babbler	0	0	0	-	-	-	X	LC
*Malacopteronalbogulare*	Grey-breasted Babbler	0	0	0	-	-	-	X	NT
*Malacocinclasepiaria*	Horsfield's Babbler	0	0	0	X	-	X	-	LC
*Pellorneumcapistratum*	Rufous-browed Babbler	0	0	0	-	-	X	X	LC
*Pellorneummalaccense*	Short-tailed Babbler	1	3	0	X	X	-	-	NT
*Pellorneumnigrocapitatum*	Black-capped Babbler	0	1	0	-	-	-	-	LC
*Malacopteroncinereum*	Scaly-crowned Babbler	0	1	0	-	-	-	X	LC
*Malacopteronmagnirostre*	Moustached Babbler	0	0	0	-	-	X	X	LC
*Pellorneumrostratum*	White-chested Babbler	1	0	0	-	-	-	-	NT
*Pellorneumtickelli*	Buff-breasted Babbler	0	0	0	-	-	-	-	LC
Phylloscopidae									
*Phylloscopusborealis*	Arctic Warbler	0	0	0	-	-	X	-	LC
*Phylloscopuscoronatus*	Eastern Crowned Warbler	0	0	0	-	-	X	-	LC
Pycnonotidae									
*Alophoixusbres*	Brown-cheeked Bulbul	0	0	0	-	-	X	-	NT
*Brachypodiusatriceps*	Black-headed Bulbul	0	3	0	-	X	X	X	LCLC
*Iolecharlottae*	Buff-vented Bulbul	0	6	0	-	X	X	-	NT
*Iolepropinqua*	Grey-eyed Bulbul	0	2	0	-	-	-	-	LC
*Pycnonotussimplex*	Cream vented Bulbul	5	5	0	-	-	X	-	LC
*Ixidiacyaniventris*	Grey-bellied Bulbul	0	1	0	X	-	X	-	NT
*Ixosmalaccensis*	Streaked Bulbul	0	0	0	-	-	X	-	NT
*Ixosmcclellandii*	Mountain Bulbul	0	0	0	-	-	-	-	LC
*Brachypodiuspriocephalus*	Grey-headed Bulbul	1	4	0	-	-	-	-	NT
*Alophoixustephrogenys*	Grey-cheeked Bulbul	0	2	0	-	-	-	X	VU
*Tricholestescriniger*	Hairy-backed Bulbul	8	3	1	X	X	X	X	LC
*Alophoixusochraceus*	Ochraceous Bulbul	0	1	0	-	-	-	-	LC
*Pycnonotusplumosus*	Olive-winged Bulbul	1	5	1	-	X	-	-	LC
*Euptilotuseutilotus*	Puff-backed Bulbul	0	1	0	-	-	-	-	NT
*Hemixosflavala*	Ashy Bulbul	0	0	0	-	X	X	X	LC
*Pycnonotusbrunneus*	Red-eyed Bulbul	2	1	1	-	X	X	X	LC
*Pycnonotusfinlaysoni*	Stripe-throated Bulbul	0	0	0	X	X	-	-	LC
*Pycnonotuspallidus*	Puff-throated Bulbul	0	0	0	-	-	-	-	LC
*Pycnonotuszeylanicus*	Straw-headed Bulbul	0	0	0	-	-	-	X	CR
*Ixidiaerythropthalmos*	Spectacled Bulbul	1	2	0	X	X	X	X	LC
*Ixidiasquamata*	Scaly-breasted Bulbul	0	0	0	-	X	X	-	NT
*Alophoixusphaeocephalus*	Yellow-bellied Bulbul	0	12	1	X	-	X	X	LC
*Rubigulamelanictera*	Black-capped Bulbul	0	0	0	-	X	X	-	LC
Rhipiduridae									
*Rhipiduraperlata*	Spotted Fantail	0	0	0	-	-	-	X	LC
*Rhipiduraalbicollis*	White-throated Fantail	0	0	0	-	-	-	-	LC
Scotocercidae									
*Abroscopussuperciliaris*	Yellow-bellied Warbler	0	0	1	-	-	X	-	LCLC
*Sittidae*									
*Sittafrontalis*	Velvet-fronted Nuthatch	0	0	0	-	-	X	-	LC
Stenostiridae									
*Culicicapaceylonensis*	Grey-headed Canary-flycatcher	0	2	0	X	-	-	-	LC
Sturnidae									
*Aplonispanayensis*	Asian Glossy Starling	0	1	0	-	-	-	-	LC
*Graculareligiosa*	Common Hill Myna	0	0	0	-	X	X	X	LC
Timaliidae									
*Erythrogenyshypoleucos*	Large-scimitar Babbler	0	0	0	-	-	-	-	LC
*Pomatorhinusschisticeps*	White-browed Scimitar-babbler	0	0	0	-	-	-	-	LC
*Stachyrisnigricollis*	Black-throated Babbler	1	0	0	-	-	-	-	NT
*Stachyrismaculata*	Chestnut-rumped Babbler	1	0	0	-	-	X	X	NT
*Stachyrisnigriceps*	Grey-throated Babbler	0	0	0	-	-	-	X	LC
*Cyanodermaerythropterum*	Chestnut winged Babbler	2	3	0	-	-	X	-	LC
*Macronusptilosus*	Fluffy-backed Tit-babbler	1	2	0	-	X	X	X	NT
*Stachyrispoliocephala*	Grey-headed Babbler	0	0	4	X	-	X	X	LC
*Mixornisgularis*	Pin-striped Tit-babbler	1	0	0	X	X	X	X	LC
Vangidae									
*Philentomapyrhoptera*	Rufous-winged Philentoma	0	2	2	-	-	X	X	LC
*Hemipuspicatus*	Bar-winged Flycatcher-shrike	0	0	0	-	-	X	-	LC
*Hemipushirundinaceus*	Black-winged Flycatcher-shrike	0	0	0	-	X	X	-	LC
*Tephrodornisvirgatus*	Large Woodshrike	0	0	0	-	X	X	-	LC
Vireonidae									
*Erporniszantholeuca*	White-bellied Erpornis	0	0	0	-	-	X	X	LC
Zosteropidae									
*Zosteropseveretti*	Everett's White-eye	0	0	0	-	X	-	-	LC
Piciformes									
Indicatoridae									
*Indicatorarchipelagicus*	Malaysian Honeyguide	0	0	0	-	-	-	X	NT
Megalaimidae									
*Psilopogonchrysopogon*	Gold-whiskered Barbet	0	0	0	-	X	X	-	LC
*Psilopogonmystacophanos*	Red-throated Barbet	0	0	0	-	-	X	-	NT
*Psilopogonaustralis*	Blue-eared Barbet	0	0	0	-	X	X	-	LC
*Caloramphusfuliginosus*	Brown Barbet	0	0	0	-	X	X	-	LC
Picidae									
*Blythipicusrubiginosus*	Maroon Woodpecker	0	0	0	X	X	X	X	LC
*Blythipicuspyrrhotis*	Bay Woodpecker	0	0	0	-	-	-	-	LC
*Chrysophlegmamentale*	Checker-throated Woodpecker	0	0	0	-	-	-	-	NT
*Chrysophlegmaminiaceum*	Banded Woodpecker	0	0	0	-	X	X	-	LC
*Chrysocolaptesvalidus*	Orange-backed Woodpecker	0	0	1	-	-	-	-	LC
*Hemicircusconcretus*	Red-crested Woodpecker	0	0	0	-	-	X	-	LC
*Hemicircussordidus*	Grey-and-buff Woodpecker	0	0	0	X	-	-	-	LC
*Meiglyptestukki*	Buff-necked Woodpecker	0	0	0	X	-	X	-	NT
*Meiglyptestristis*	White-rumped Woodpecker	0	0	0	-	X	X	-	EN
*Micropternusbrachyurus*	Rufous Woodpecker	0	0	0	X	X	X	-	LC
*Picuspuniceus*	Crimson-winged Woodpecker	0	0	0	-	-	X	-	LC
*Sasiaabnormis*	Rufous Piculet	0	0	0	-	-	X	X	LC
Psittaciformes									
Psittacidae									
*Loriculusgalgulus*	Blue-crowned Hanging-parrot	0	0	0	-	X	X	-	LC
Strigiformes									
Strigidae									
*Bubosumatranus*	Barred Eagle-owl	0	1	0	-	-	X	-	LC
*Otuslettia*	Collared Scops-owl	0	3	0	-	-	-	-	LC
*Otussunia*	Oriental Scops-owl	0	1	0	-	-	-	-	LC
*Otusbakkamoena*	Indian Scops-owl	0	0	0	-	X	X	X	LC
*Otusspilocephalus*	Mountain Scops-owl	0	0	0	-	-	-	-	LC
*Phodilusbadius*	Oriental Bay-owl	0	0	0	-	-	-	X	LC
Trogoniformes									
Trogonidae									
*Harpactesdiardii*	Diard's Trogon	0	0	0	-	-	X	-	NT
*Harpactesduvaucelii*	Scarlet-rumped Trogon	0	0	0	-	-	X	X	NT
*Harpacteskasumba*	Red-naped Trogon	0	0	0	-	-	-	-	NT
	Total Individuals	54	131	40					

**Table 4.** Diversity of understorey birds in three selected environmentally sensitive areas of permanent forest reserves in northern region of Selangor.

**Sites**	**Relative Abundance (%)**	**Richness (S)**	**Shannon (H')**	**Dominance (D)**	**Evenness**	**Chao 1**
**BTE**	24	33	3.24	0.054	0.774	79
**GFR**	58.2	46	3.43	0.047	0.673	69.3
**BKFR**	17.8	21	2.84	0.071	0.815	34.2

**Table 5.** SIMPER percentage (%) contribution of dominant bird species at various levels of disturbances.

**Taxon**	**Av. dissim**	**Contrib.** %	**Cumulative** %
Fluffy-backed tit babbler (*Macronusptilosus*)	20.84	25.45	25.45
Fiery Minivet (*Pericrocotusigneus*)	13.73	16.77	42.22
Yellow-bellied warbler (*Abroscopussuperciliaris*)	13.7	16.73	58.95
Tiger shrike (*Laniustigrinus*)	0.3429	0.4187	59.37
White-breasted Waterhen (*Amaurornisphoenicurus)*	0.3177	0.3879	59.75
Chestnut-naped Forktail (*Enicurusruficapillus*)	0.2875	0.3511	60.11
